# Spatiotemporal remodelling of the cloacal region determines the position of the anal opening in mouse embryos

**DOI:** 10.1111/joa.70110

**Published:** 2026-02-03

**Authors:** Weiyi Wang, Masayo Harada, Keiichi Akita

**Affiliations:** ^1^ Department of Clinical Anatomy Graduate School of Medical and Dental Sciences, Institute of Science Tokyo Bunkyo‐ku Tokyo Japan

**Keywords:** anal opening, cloaca, cloacal membrane, hindgut, urogenital sinus, urorectal septum

## Abstract

Conventional embryological models propose that the urorectal septum (URS) fuses with the cloacal membrane (CM) to form separate exits for the hindgut and urogenital sinus (UGS). However, previous studies have been re‐evaluating this assumption, and the precise morphogenetic mechanisms determining the position of the anal opening remain unclear. To elucidate the spatial and temporal dynamics of cloacal development, we analysed mouse embryos from embryonic day (E)11.5 to E13.5 using serial midsagittal histological sections combined with three‐dimensional reconstruction. We focused on the positional relationship between the URS and CM, as well as the internal remodelling events of the cloaca that lead to anal opening formation. Throughout development, the URS enlarged and shifted distally, but consistently remained dorsodistal to the CM without direct fusion. At E13.0, we identified an expanded space at the caudal end of the hindgut, distinct from the hindgut lumen. By E13.25, this space connected to the UGS via a duct‐like structure, contributing to the separation of the UGS and hindgut. By E13.5, the CM ruptured and the anal opening emerged precisely at the junction between the hindgut lumen and the expanded space. Our findings demonstrate that the position of the anal opening is predetermined by cloacal internal space remodelling rather than fusion of the URS and CM. This study offers novel insights into normal anorectal development and the aetiology of congenital anorectal malformations.

## Graphical abstract

In E13.25 mouse embryo, the urorectal septum consistently positioned on the dorsodistal side of the cloacal membrane. Through 3D reconstruction of the cloacal internal space, we revealed that the position of the anal opening is established prior to cloacal membrane rupture and is located at the junction between the hindgut lumen (blue) and an expanded space at the caudal end of the hindgut (red).

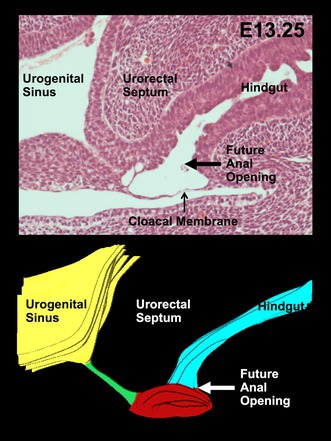

## 1 INTRODUCTION

The formation and remodelling of the urorectal septum (URS) and the cloacal membrane (CM) are key features of cloacal septation. The classical models proposed by Rathke and Tourneux, based on two‐dimensional (2D) histological observations, were once widely accepted. Rathke suggested that bilateral folds from the lateral cloacal walls fuse at the midline to form the septum (Rathke, 1832), whereas Tourneux proposed that the septum arises from the dorsal cloacal wall and elongates caudally to divide the cloacal lumen (Tourneux, 1888). Although these models differ in the proposed origin of the septum, both imply an active role of septal descent and its fusion with the CM in separating the hindgut (Hg) from the urogenital sinus (UGS).

However, some studies have reported no direct fusion between the URS and the CM (Kluth et al., 1995; Kluth et al., 2011), suggesting that the septum may play a lesser role in CM rupture than previously assumed by Rathke and Tourneux. Moreover, the morphological changes of the cloacal internal space—particularly the relationship between the URS and CM dynamics—remain unclear.

In this study, we used serial histological sectioning and three‐dimensional (3D) reconstruction to investigate cloacal development in mouse embryos from E11.5 to E13.5. We aimed to clarify the positional relationship between the URS and CM, and to identify the morphogenetic processes involved in CM rupture and anal opening formation. By combining conventional histology with 3D reconstruction, we provide new insights into cloacal development, contributing to a more accurate and updated understanding of anorectal morphogenesis.

## 2 METHODS

### 2.1 Animals and histological staining

Pregnant Jcl:ICR mice were purchased from CLEA Japan, Inc. To assess the sequential development of the cloacal region, two pregnant mice were sacrificed at E11.5, E12.0, E12.25, E12.5, E12.75, E13.0, E13.25 and E13.5, and 20 embryos were collected and analysed at each stage. All animal experiments were conducted in accordance with institutional guidelines for animal care and were approved by the Institutional Animal Care and Use Committee of the Institute of Science Tokyo (Approval date: 1 April 2025; research number: A2025‐010A).

Embryos were collected in ice‐cold phosphate‐buffered saline (PBS) and fixed overnight in 4% paraformaldehyde in PBS at 4°C. They were then dehydrated in methanol and embedded in paraffin wax. Serial midsagittal sections (5 μm thick) were prepared and stained with haematoxylin and eosin (H&E).

### 2.2 3D reconstruction

3D reconstructions of the cloaca were generated from H&E‐stained serial midsagittal paraffin sections of embryos at E12.75 to E13.5. Images were captured using a microscope (ECLIPSE E400; Nikon) equipped with a digital camera (DS‐Fi1; Nikon). The contours of the cloacal internal space were traced manually. Image stacks were aligned and reconstructed using the SrfII software (version R.11.00.00.0‐H; Ratoc Systems Engineering, Tokyo, Japan), enabling volumetric visualisation and spatial analysis of cloacal morphology.

## 3 RESULTS

### 3.1 Ventrodistal movement of the Hg and formation of the CM

**FIGURE 1:**
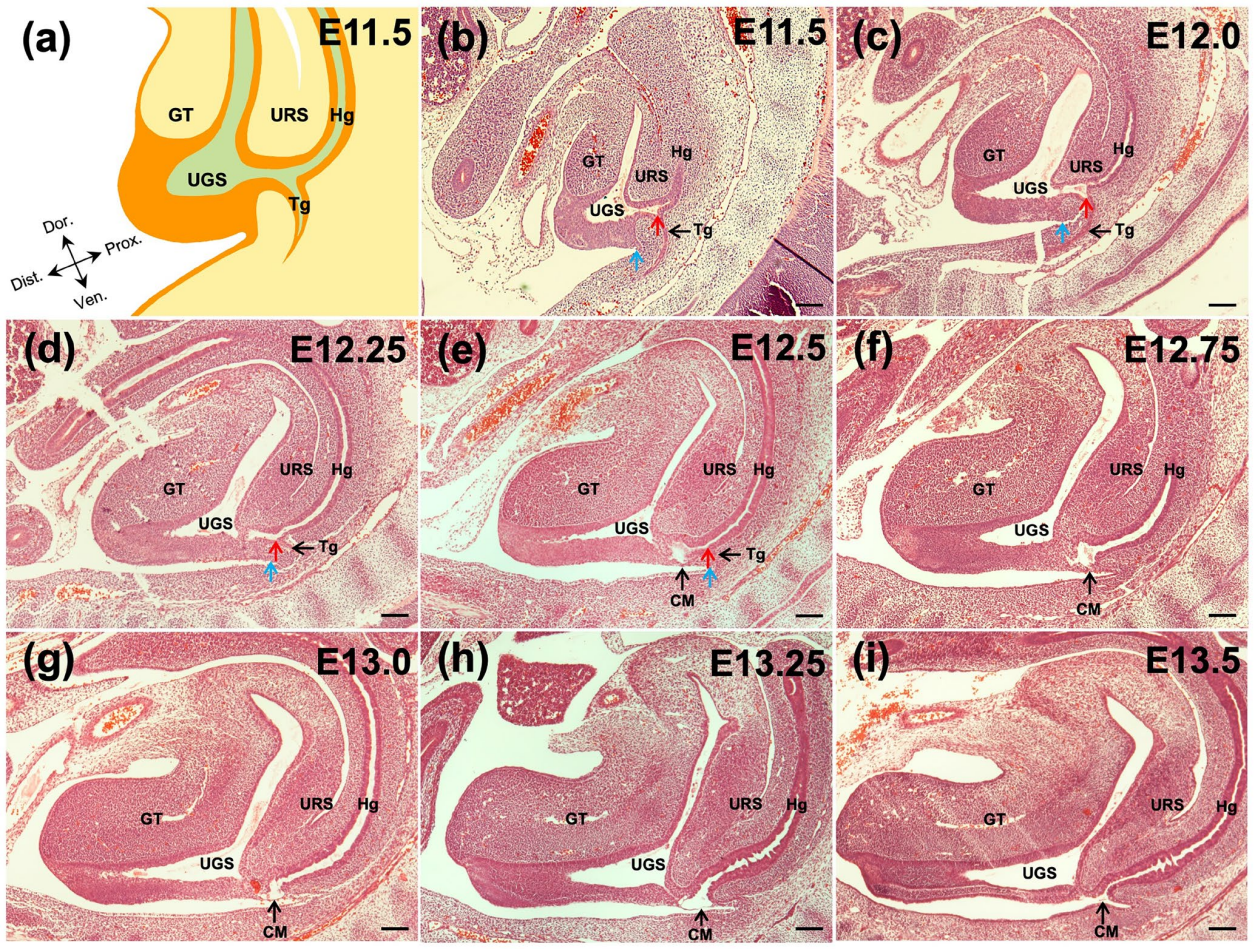
Cloacal development from E11.5 to E13.5. (a) Schematic diagram of the cloaca indicating anatomical orientation. (b–i) H&E‐stained midsagittal sections of the cloaca at E11.5 (b), E12.0 (c), E12.25 (d), E12.5 (e), E12.75 (f), E13.0 (g), E13.25 (h) and E13.5 (i). The Hg moved ventrodistally and the CM formed during this period. Red arrow, tailgut entrance; blue arrow, bottom starting point of the genital tubercle; CM, cloacal membrane; GT, genital tubercle; Hg, hindgut; Tg, tailgut; UGS, urogenital sinus; URS, urorectal septum. Scale bars: 0.1 mm.

A schematic diagram of the cloaca with directional orientation is shown in Figure 1a. We examined normal cloacal development sequentially using sagittal sections of mouse embryos from E11.5 to E13.5 (Figure 1b–i). At each developmental stage, 20 embryos were analysed, and no notable interindividual differences in cloacal morphology were observed among them. Cloacal development started before E11.5 (Figure 1b). From E11.5 to E13.5, the genital tubercle developed and grew substantially, and the URS enlarged and moved distally towards the ventral cloacal epithelium (Figure 1b–i). At E13.5, the cloaca was completely divided into the UGS and Hg (Figure 1i).

At E11.5 and E12.0, no clear boundary was observed between the UGS and Hg. The tailgut opened into the posterior wall of the Hg—referred to as the tailgut entrance (red arrow)—which was located proximal to the starting point of the genital tubercle (blue arrow) (Figure 1b,c).

At E12.25, the ventrodistal portion of the URS had descended, establishing a boundary between the distal and proximal regions of the cloaca. The tailgut entrance (red arrow) moved ventrodistally and was now closer to the bottom starting point of the genital tubercle (blue arrow) compared with earlier stages (Figure 1d).

At E12.5, the tailgut entrance (red arrow) moved further ventrodistally. Simultaneously, the proximal region of the ventral cloacal epithelium underwent marked degeneration and thinning, forming the CM (Figure 1e).

At E12.75, the tailgut had completely degenerated, and the CM epithelium became thinner (Figure 1f). At E13.0, the CM comprised a single layer of epithelial cells (Figure 1g). At E13.25, the CM extended distally (Figure 1h). At E13.5, the CM ruptured, and the cloaca became connected to the external space (Figure 1i).

In summary, the Hg underwent a ventrodistal shift from E11.5 to E12.5, and the CM first became clearly identifiable at E12.5.

### 3.2 Positional relationship between the URS and the CM

**FIGURE 2:**
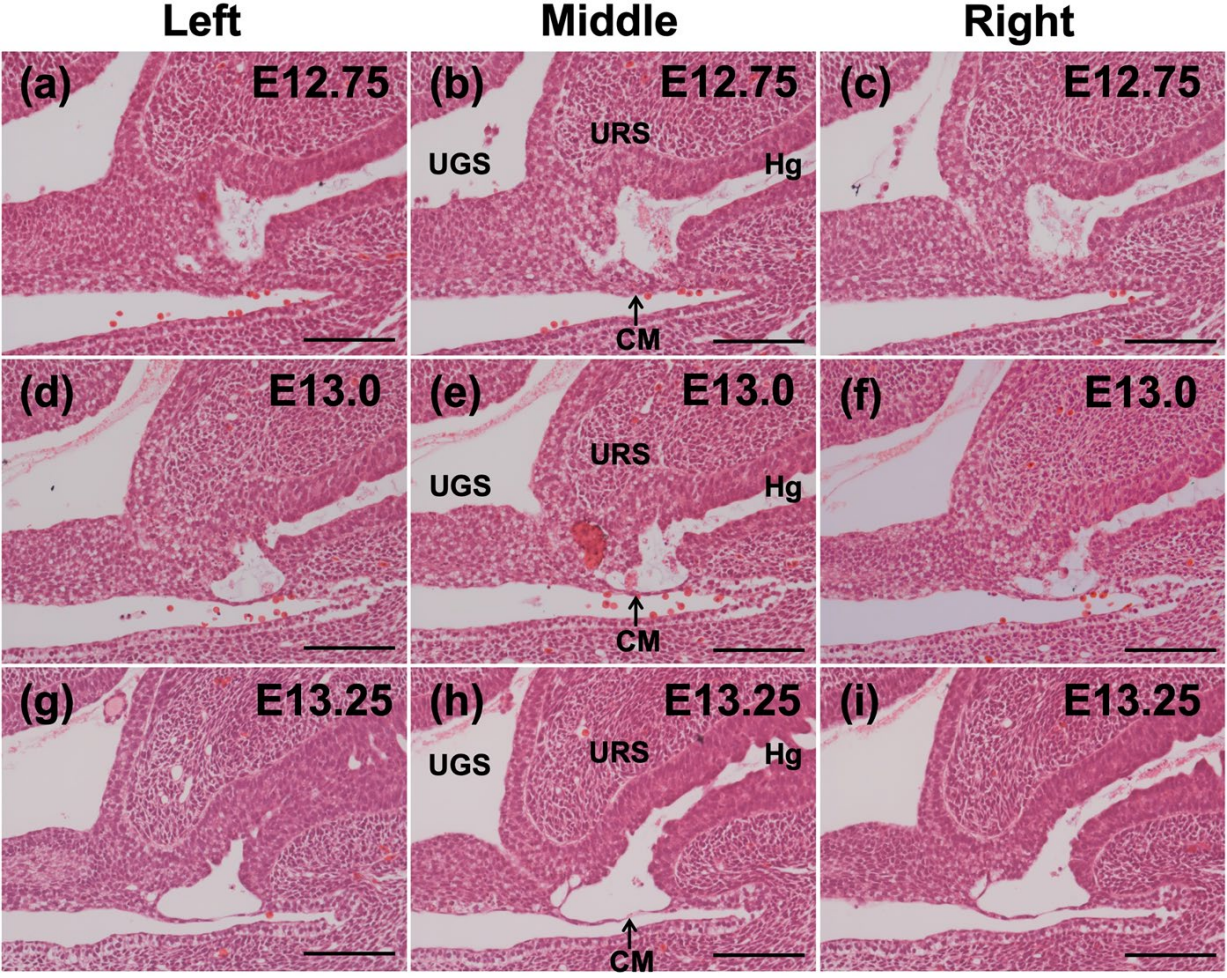
Positional relationship between the URS and CM from E12.75 to E13.25. H&E‐stained sagittal sections at E12.75 (a–c), E13.0 (d–f), E13.25 (g–i). The middle panels (b, e, h) show midsagittal sections, with adjacent left (a, d, g) and right (c, f, i) sagittal sections at each stage. The URS enlarged and extended distally but remained located dorsodistally relative to the CM throughout. CM, cloacal membrane; Hg, hindgut; UGS, urogenital sinus; URS, urorectal septum. Scale bars: 0.1 mm.

To examine the positional relationship between the URS and the CM, we analysed midsagittal and adjacent left and right sagittal sections from E12.75 to E13.25 (Figure 2).

At E12.75, the proximal region of the ventral cloacal epithelium was thinner than the distal region (Figure 2a–c). At this stage, the URS had descended to a position near the epithelial tissue of the CM in the midsagittal section (Figure 2b). At E13.0, the CM became a single‐layer epithelium. The URS did not descend further but shifted distally, coming into contact with the ventral cloacal epithelium (Figure 2d–f). At E13.25, the CM had extended further distally, while the URS remained dorsal and distal to it (Figure 2g–i). In the midsagittal section, the caudal end of the URS was separated from the ventral cloacal epithelium (Figure 2h).

In summary, the URS consistently remained dorsal and distal to the CM throughout the stages examined, without direct interaction, from the onset of CM formation to its rupture.

### 3.3 Separation of the UGS exit from the Hg lumen before CM rupture

We examined the temporal changes in the shape of the cloacal internal space from E12.75 to E13.5 with finer temporal resolution (Figure S1).

**FIGURE 3:**
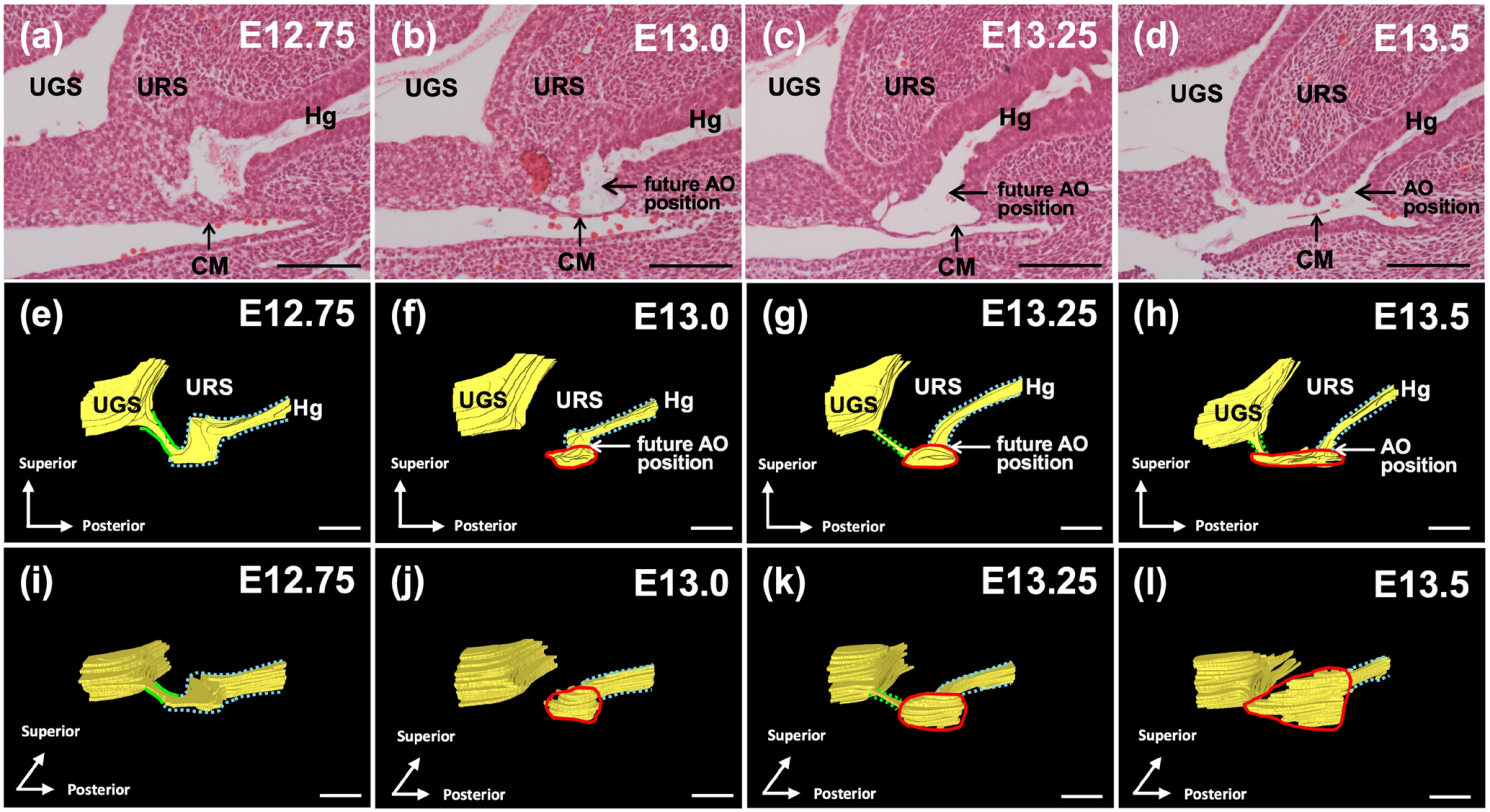
Three‐dimensional (3D) reconstructions of the cloaca from E12.75 to E13.5. H&E‐stained midsagittal sections of the cloaca (a–d). Sagittal views (e–h) and low‐angle horizontal views (i–l) of 3D reconstructions of the cloacal internal space. The UGS exit separated from the hindgut lumen before CM rupture. The anal opening was formed at the junction between the hindgut lumen and an expanded space. Green line, connection between UGS and hindgut lumen; blue dotted line, hindgut lumen; red line, expanded space at the caudal end of the Hg; green dotted line, duct‐like space; AO, anal opening; CM, cloacal membrane; Hg, hindgut; UGS, urogenital sinus; URS, urorectal septum. Scale bars: 0.1 mm.

At E12.75, although the URS appeared to be partially in contact with the ventral cloacal epithelium in the midsagittal section (Figure 3a), a connection (green line) between the UGS and the Hg lumen (blue dotted lines) was still present in the 3D reconstruction of the cloacal internal space (Figure 3e).

At E13.0, the disorganised epithelium of the URS was loosely connected to the ventral cloacal epithelium in the midsagittal section (Figure 3b). In the 3D reconstruction, the connection between the UGS and the Hg had closed, resulting in two separate compartments. At this stage, an expanded space appeared at the caudal end of the Hg (red line), which was located adjacent to the CM (Figure 3b,f).

At E13.25, the CM had extended distally and the ventral cloacal epithelium separated from the URS in the midsagittal section (Figure 3c). The 3D reconstruction revealed a narrow, elongated connection (green dotted lines) between the UGS and the expanded space at the caudal end of the Hg (red line). This newly formed duct‐like space (green dotted lines) was situated between the URS and the ventral cloacal epithelium (Figure 3g). Notably, this duct‐like space did not directly connect with the Hg lumen (blue dotted lines), indicating complete separation of the UGS exit (green dotted lines) from the Hg lumen (blue dotted lines) (Figure 3g).

At E13.5, the CM ruptured, forming a connection between the cloacal and external spaces (Figure 3d). The 3D reconstruction indicated that the duct‐like space shortened as the URS moved further distally (Figure 3h).

These findings suggest that the UGS exit was separated from the Hg lumen prior to CM rupture.

### 3.4 Cell death associated with the UGS exit formation

**FIGURE 4:**
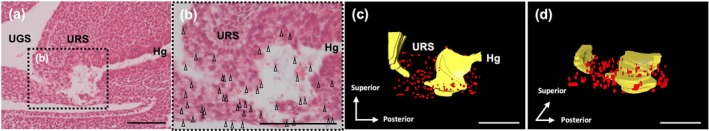
Cell death associated with the UGS exit formation at an approximate developmental stage between E13.0 and E13.25. H&E‐stained midsagittal sections of cloaca (a, b). Sagittal view (c) and low‐angle horizontal view (d) of three‐dimensional (3D) reconstruction of the cloacal internal space (yellow) and epithelial cell death (red spots). Arrowheads, epithelial cell death; Hg, hindgut; UGS, urogenital sinus; URS, urorectal septum. Scale bars: 0.1 mm.

We examined the cellular properties associated with the UGS exit formation immediately before the emergence of the UGS exit (Figure 4).

In the midsagittal section, we observed epithelial cell death (arrowheads in Figure 4b) between the UGS and Hg, particularly in the area where the disorganised epithelium of the distal URS was loosely connected to the ventral cloacal epithelium. 3D reconstruction revealed epithelial cell death (red spots in Figure 4c,d) adjacent to the boundary between the distal URS and the ventral cloacal epithelium, prior to UGS exit formation (green dotted lines in Figure 3g).

These observations suggest that epithelial cell death contributes to the remodelling of the cloacal internal space, facilitating the separation of the UGS exit from the hindgut lumen.

### 3.5 Morphological changes of cloacal internal space and the location of the anal opening

At E13.0, 3D reconstruction revealed an expanded space at the caudal end of the Hg (red line) (Figure 3f). When viewed from a low‐angle perspective, this space had a greater horizontal diameter than the Hg lumen (Figure 3j).

By E13.25, the expanded space at the caudal end of the Hg (red line) had widened further, accompanied by the continued expansion of the CM (Figure 3c,g,k). At E13.25, both the duct‐like space (green dotted lines) and the Hg lumen (blue dotted line) opened into the expanded space at the caudal end of the Hg (red line) (Figure 3g). Finally, by E13.5, the CM ruptured and the anal opening—serving as the exit of the Hg lumen (white arrow in Figure 3h)—was positioned at the junction between the Hg lumen (blue dotted line) and the expanded caudal space (red line) (Figure 3d,h,l).

## 4 DISCUSSION

Although previous studies have demonstrated that morphological changes of the URS and the formation and rupture of the CM play important roles in anal formation process, little attention has been paid to the changes of the cloacal internal space during cloaca development. In this study, we analysed morphological changes in the cloacal internal space and the positioning of the anal opening using histological sectioning and 3D reconstruction techniques.

We found that the ventral cloacal epithelium became significantly thinner at the future position of the CM, accompanied by distal elongation of the genital tubercle and ventrodistal movement of the Hg. Previous studies have demonstrated that apoptosis plays essential roles in normal cloacal development, particularly in the remodelling of the ventral cloacal epithelium (Miyagawa et al., 2014; Sasaki et al., 2004; Seifert et al., 2009). In addition to these findings, 3D analyses of cloacal epithelial dynamics have also been reported (Matsumaru et al., 2015). It is conceivable that the initial formation of the space through apoptosis was followed by progressive CM epithelial thinning, which reduced tissue tension and passively allowed the space to expand. These changes occurred in parallel with URS morphological change and distal movement, contributing to the formation of the expanded space at the caudal end of the Hg (red line in Figure 3f,j) observed at E13.0 and the duct‐like space (green dotted lines in Figure 3g,k) at E13.25. This expanded space facilitated separation of the UGS exit from the Hg lumen. Notably, the position of the anal opening is at the junction of the Hg lumen and this expanded space, which is determined before the CM rupture. Regarding the duct‐like space, we consider that this space is the embryonic origin of the urethra. This interpretation is supported by the previous research showing that UGS endoderm directly contributes to the lower urinary outlet during normal cloacal subdivision (Seifert et al., 2008).

Classical models by Rathke and Tourneux suggested that the URS eventually fuses with the CM, resulting in CM rupture and the formation of the anal and UGS openings, based on coronal and sagittal sections, respectively (Rathke, 1832; Tourneux, 1888). Some authors have supported the idea that URS–CM fusion is a key step in cloacal opening formation (Bai et al., 2004; Gupta et al., 2014; Qi et al., 2000; Zhang et al., 2011), whereas others claim that the URS does not actively descend towards the CM and that fusion does not occur (Kluth et al., 1995; Kluth et al., 2011; Nievelstein et al., 1998; Sasaki et al., 2004; Van Der Putte, 2009). Regarding this controversy, we suggest that 2D analyses and high‐resolution electron microscopy are limited in their ability to capture the temporal and spatial dynamics of cloacal development. By employing 3D reconstruction, we provide a more comprehensive and spatially accurate view of cloacal development. In our study, we observed that the CM formed at E12.75, and that the URS remained consistently dorsal and distal to the CM throughout development. Based on 3D reconstruction of the spatiotemporal dynamics of the cloaca, we demonstrated that before E13.0, the connection between the UGS and the Hg lumen constituted a structure linking the distal and proximal spaces of the cloaca, and this connection closed at E13.0. At E13.25, a duct‐like space subsequently opened between the UGS and the expanded space at the caudal end of the Hg in the cloaca. These findings indicate that the URS and CM undergo distinct morphogenetic processes. Therefore, we propose that CM rupture is not caused by fusion with the URS, but is instead a consequence of cloacal internal space remodelling and spontaneous disruption of the CM.

The spatial determination of the anal opening has been interpreted in divergent ways. Some studies suggest that the anal opening forms just proximal to the fusion point between the URS and CM (Bai et al., 2004; Gupta et al., 2014; Qi et al., 2000; Zhang et al., 2011). In contrast, others have reported that the URS and CM remain anatomically distinct throughout development, suggesting that the anal opening arises at a fixed point near the proximal end of the CM, identifiable from early developmental stages (Kluth et al., 1995; Kluth et al., 2011; Nievelstein et al., 1998; Van Der Putte, 2009). Our study supports the hypothesis that the anal opening forms at a fixed position serving as the exit of the Hg lumen. However, our results revealed that this position is not located on the CM. Instead, we show—using 3D reconstruction—that the anal opening is established before CM rupture, at the junction between the Hg lumen and the expanded space at the caudal end of the Hg, which emerges around E13.0. These findings indicate that the position of the anal opening is not determined by the CM itself, but rather by earlier internal remodelling events within the cloaca.

It should be noted that our conclusions are based on observations in mouse embryos. Previous study in humans (Zhang et al., 2011) has suggested that URS–CM fusion is a key step in cloacal opening formation. Such discrepancies may reflect species‐specific differences in the morphology of Hg development. Comparative analysis across species will be necessary.

In summary, our findings emphasise the critical role of cloacal internal spatial remodelling in determining the anal opening position. A limitation of this study is that it focuses primarily on morphological observations. Future studies should clarify the molecular mechanisms, cellular behaviours and tissue interactions involved in epithelial rearrangement and spatial remodelling of the cloaca. Investigating how cloacal space remodelling is induced may bridge the gap between anatomical observations and developmental mechanisms, ultimately advancing our understanding of the aetiology of anorectal malformations.

## 5 CONCLUSIONS

This study showed that the URS and CM remain dynamically and spatially separated during cloacal development, with the URS consistently positioned on the dorsodistal side of the CM. Through 3D reconstruction of the cloacal internal space, we showed that the position of the anal opening is established prior to CM rupture and is located at the junction between the Hg lumen and an expanded space at the caudal end of the Hg. These findings highlight the significance of internal space remodelling in cloacal development and provide new insight into the mechanisms underlying anal opening formation.

## AUTHOR CONTRIBUTIONS

W. W. carried out the data acquisition, analysis, interpretation and manuscript drafting. M. H. conducted the study conception and design, data interpretation, critical revision of the manuscript and approval of the final version. K. A. was involved in the study conception and design, data interpretation, critical manuscript revision and approval of the final version for publication.

## CONFLICT OF INTEREST STATEMENT

The authors declare no conflicts of interest, financial or otherwise.

## Supporting information


**Figure S1.** Detailed 3D reconstructions of the cloaca from E12.75 to E13.5. H&E‐stained sagittal sections and 3D reconstructions from E12.75 to E13.5 showing the progressive remodelling of the cloacal internal space from younger to older stages. CM, cloacal membrane; Hg, hindgut; UGS, urogenital sinus; URS, urorectal septum. Scale bars: 0.1 mm.

## Data Availability

The data that support the findings of this study are available on request from the corresponding author. The data are not publicly available due to privacy or ethical restrictions.
