# Supplementary figures and images for: Spatiotemporal remodelling of the cloacal region determines the position of the anal opening in mouse embryos
Source: J Anat. 2026 Feb 3;249(3):652–8. doi: 10.1111/joa.70110 (PMC13399002; doi:10.1111/joa.70110)

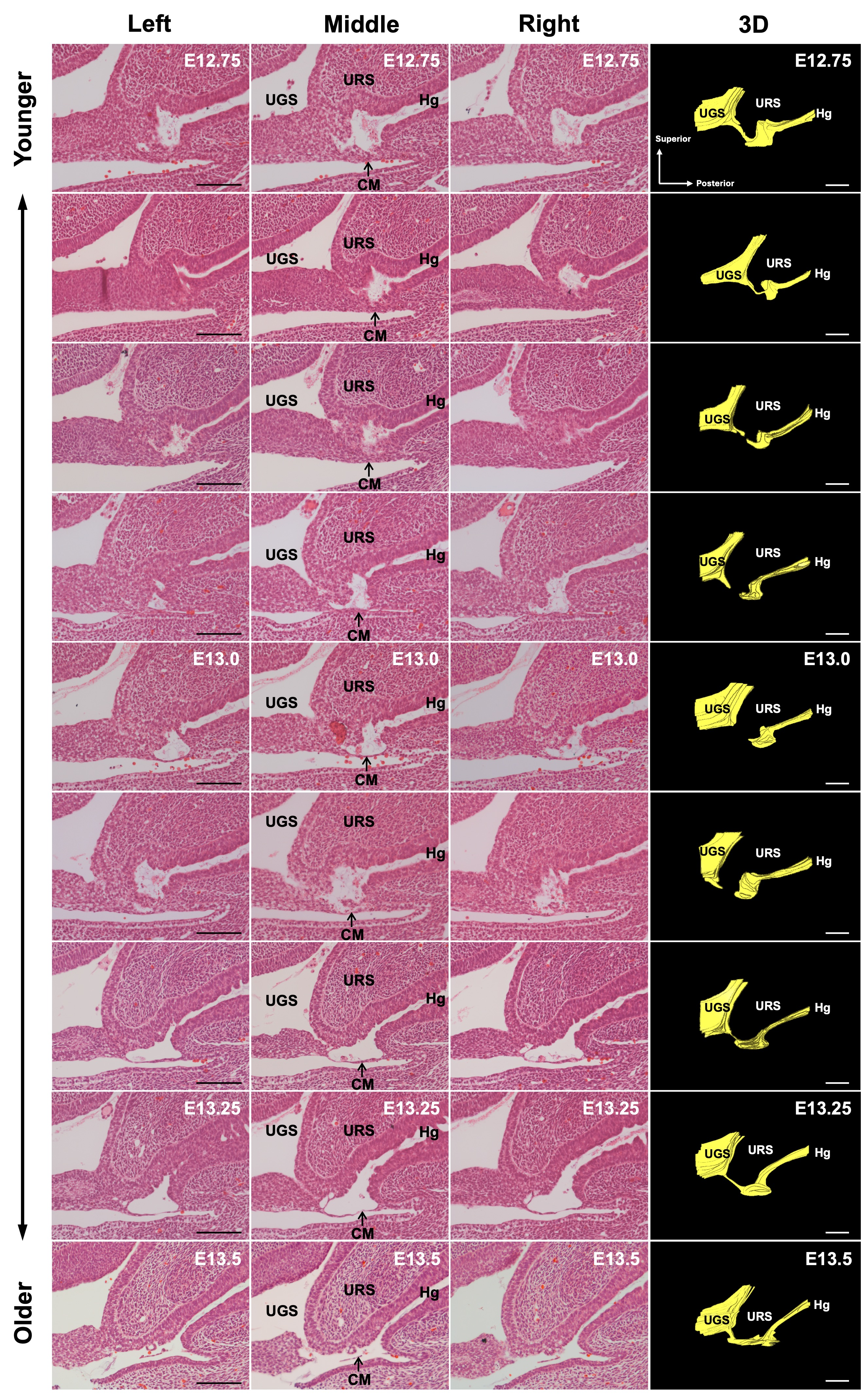

Supplement: Supplementary file 1 — Figure S1. Detailed 3D reconstructions of the cloaca from E12.75 to E13.5. H&E‐stained sagittal sections and 3D reconstructions from E12.75 to E13.5 showing the progressive remodelling of the cloacal internal space from younger to older stages. CM, cloacal membrane; Hg, hindgut; UGS, urogenital sinus; URS, urorectal septum. Scale bars: 0.1 mm. [file JOA-249-652-s001.tiff]
